# Real world data on digital remote refraction in a healthy population of 14,680 eyes

**DOI:** 10.1038/s41746-025-01453-0

**Published:** 2025-02-06

**Authors:** Casper van der Zee, Heshow Jamal, Marc Muijzer, Laurence Frank, Gerko Vink, Robert Wisse

**Affiliations:** 1 Ophthalmology Department, University Medical Center Utrecht, Utrecht, The Netherlands; 2 Easee B.V., Amsterdam, The Netherlands; 3 Department of Methodology and Statistics, Faculty of Social and Behavioural Sciences, Utrecht University, Utrecht, The Netherlands

**Keywords:** Refractive errors, Health services, Eye manifestations, Medical research, Physical examination

## Abstract

Refractive errors are the leading cause of preventable visual impairment, to which web-based remote refraction could contribute. We report real-world 2021–2022 data of the underlying algorithm and validated these to conventional prescriptions among healthy individuals (high visual acuity and satisfactied current refraction). Participants were 18–45 years with a spherical (S) error between −3.50 + 2.00S to −2.00 Diopter Cylinder (DC), reported as Spherical Equivalent (SEQ) in mean differences and 95% Limits of agreement. Consecutive measurements (*n* = 14,680) were assessed of which *n* = 6386 selected for validation. The mean difference was 0.01D(SD 0.69) and −0.73D(SD 0.92) for myopes and hyperopes respectively. This algorithm shows variation, nonetheless, 67% and 82% of myopes were within ±0.5 and ±0.75D. The test underestimates hyperopes (34% and 50% within ±0.5D and ±0.75D) and had inconsistencies distinguishing hyperopia. This proof-of-concept shows home testing has the potency to increase accessibility to care by delivering a valuable alternative for uncomplicated refractive assessments.

## Introduction

Refractive errors are the leading cause of preventable visual impairment (VI) worldwide^1,2^. For adults and children, myopia is the predominant refractive error^3^. Although the prevalence of myopia varies significantly based on a number of factors such as location and age, it is estimated half of the world’s population will have myopia by 2050^4,5^. Moreover, in 2020 between 510 and 826 million people with presbyopia are reported to have VI simply due to not wearing adequate reading glasses, also expected to increase significantly in the future^6^. Implications of VI are severe; over $205 billion is estimated in reduced productivity yearly due to refractive errors for mild, moderate, and severe VI^7^. This is compounded by lagging academic and personal development, reduced quality of life, increased mortality, and depression^1,2,8–13^. The biggest drivers for uncorrected refractive errors (URE) are population growth, environmental factors, and lack of access to health care^14,15^. Ironically, the return on investment of visual aids is roughly 10-fold on a societal perspective and is among the most cost-effective interventions in health care^6,16^. Yet, conventional in-office refractive error assessments require trained staff, specialized equipment, mydriatics, or all; whereby UREs remain a gobal public health problem due to a lack of access to eye care services, particularly in less developed regions of the world.

In this context, telemedicine could be an answer to the stressed access to care. To this end, this online eye test was developed which performs a remote web-based assessment of visual acuity and refractive errors. A major benefit of this digital test is its scalability compared to in-office refractive methods;– participants can independently perform the test if they can control a phone and a computer. The performance of the underlying algorithms was established in a 2019 clinical study and deemed non-inferior to a gold standard manifest subjective refraction by an optometrist^17^. In the commercially available online test, a trained and experienced optometrist reviews the data and user-performance prior to issuing a prescription. In this report, we focus on the algorithm performance, and the optometrist-derived prescriptions are not included. Many online eye tests are available, either web-based or on iOS/android, but as yet only a minority are clinically validated and certified as a Medical Device^18^. The validity of this test has been reported previously in controlled settings^17,19,20^. However, it is of paramount importance to assess the external validity and accuracy of this novel technology in a real-world setting since these data validate the debate on the value of remote refraction in health care.

This paper therefore reports on the real-world performance of a web-based eye test in a healthy population in an uncontrolled home-environment, in terms of validity and accuracy. Data analyses and reporting are commensurate with the existing clinical validation study by Wisse et al., expanded with bias- and subgroup analyses^17^.

## Results

### Description of study population

**Fig. 1:**
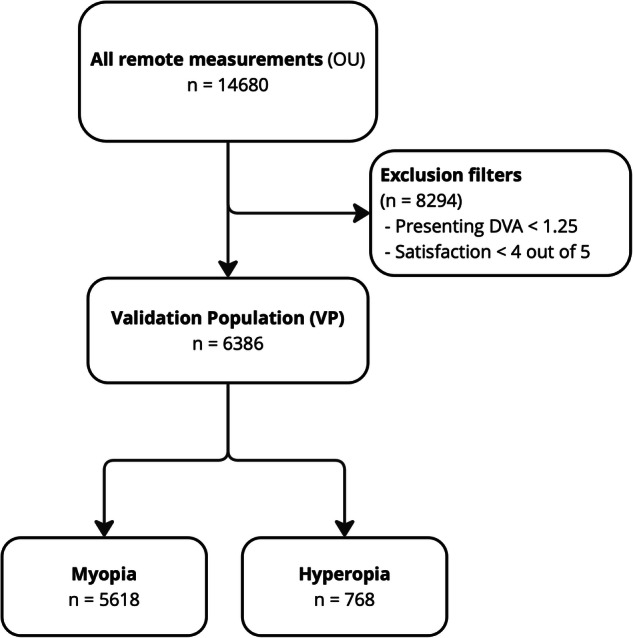
Flowchart for the validation population: (PDVA ≥ 1.25 decimal Snellen, satisfaction ≥4/5). The primary analysis reported in this paper is performed on 6386 eyes. Considering the validation of the test with the existing prescription, inclusion of only users with a high attained visual acuity was justified. The reason for a lower VA in the excluded cases could not be determined, and a faulty existing prescription was arguably a driver to perform a new eye test. Therefore these uncontrolled data were excluded for validation purposes. PDVA presenting distance visual acuity, OU oculus uterque (both eyes). Hyperopic/myopic signation as determined by the existing in-office prescription (IOP).

Measurements of both eyes (ODS; *n* = 14,680) were assessed between January 1^st^ 2021 and December 31^st^ 2022, comprising all completed consecutive refractive assessments of the online eye test. Characteristics of the complete population are reported in Table 1. Based on a high presenting distance visual acuity (PDVA; ≥1.25 Snellen, ≥20/16) and a high satisfaction with the current eyeware (4/5 or 5/5), a validation population was decided. The characteristics of the validation population (VP) are reported in Table 2 and Fig. 1. In total, 6386 measurements of the VP were eligible for analyses. At baseline, the VP did not differ materially from the complete population, yet the VP consisted of less females (52% vs 58%), slightly less hyperopes (12% vs 13%), and a slight difference in Spherical Equivalent (SEQ; −1.16D vs −1.11D).

**Table 1:** Baseline characteristics of the complete population

Variables	Overall^a^ *N* = 14,680 eyes	Myopia^a^ *N* = 12,784 eyes	Hyperopia^a^ *N* = 1896 eyes
Sex (female)^b^	8514 (58%)	7373 (58%)	1141 (60%)
Age (years)^b^	30.74 (7.10)	30.55 (6.96)	32.03 (7.91)
Eye OD, (*n*) Eye OS, (*n*)	7340 (50%) 7340 (50%)	6408 (50%) 6376 (50%)	932 (49%) 964 (51%)
PDVA^c^	1.09 (0.35)	1.09 (0.34)	1.10 (0.37)
UDVA^c^	0.43 (0.29)	0.39 (0.27)	0.68 (0.31)
Habitual IOP Sphere (D)	−0.87 (1.01)	−1.11 (0.82)	0.75 (0.57)
Habitual IOP Cylinder (D)	−0.47 (1.21)	−0.51 (0.56)	−0.25 (3.02)
Habitual IOP Axis (degrees)	76.53 (65.39)	76.05 (65.22)	79.77 (66.46)
Test time (min)	15.19 (11.14)	15.24 (11.19)	14.86 (10.75)
Concordant signation	13,321 (91%)	12,257 (96%)	832 (44%)
Satisfaction rate with IOP ≥ 4/5^b^	10,438 (72%)	9208 (73%)	1230 (66%)
Interval between IOP and online test			
<6 months	300 (19%)	279 (19%)	21 (15%)
6 months – 1 year	196 (12%)	182 (12%)	14 (10%)
1–3 years	744 (46%)	686 (46%)	58 (42%)
>3 years	380 (23%)	335 (23%)	45 (33%)

Characteristics of the total population with a refractive error between -3.50D and +2.00D sphere and ≤2.00D cylinder determined by the regulated measurement capability of the device. Ametropia (myopia/hyperopia) was determined by the conventional in-office prescription (IOP).

*D* Diopters, *OD* oculus dexter (right eye), *PDVA* presenting distance visual acuity, *UDVA* uncorrected distance visual acuity.

^a^*n* (%); Mean (SD).

^b^All numbers pertain to separate monocular measurements, with the exception of sex, age, interval between both refractions, and satisfaction rate.

^c^As measured by the web-based test.

**Table 2:** Baseline characteristics of the validation population (VP)

Variable	Overall^a^ *N* = 6386 eyes	Myopia^a^ *N* = 5618 eyes	Hyperopia^a^ *N* = 768 eyes
Sex (female)^b^	3294 (52%)	2883 (51%)	411 (54%)
Age (years)^b^	30.80 (6.80)	30.75 (6.68)	31.18 (7.59)
Eye (OD)	3193 (50%)	2814 (50%)	379 (49%)
PDVA^c^	1.31 (0.27)	1.31 (0.27)	1.32 (0.30)
UDVA^c^	0.49 (0.32)	0.45 (0.30)	0.77 (0.31)
Habitual IOP Sphere (D)	−0.91 (1.01)	−1.14 (0.83)	0.75 (0.56)
Habitual IOP Cylinder (D)	−0.49 (0.56)	−0.51 (0.54)	−0.35 (0.69)
Habitual IOP Axis (degrees)	76.87 (65.22)	76.34 (64.92)	80.76 (67.25)
Test time (min)	15.23 (11.17)	15.24 (11.11)	15.11 (11.60)
Concordant signation	5822 (91%)	5390 (96%)	336 (44%)
Satisfaction rate with IOP ≥ 4/5^b^	6386 (100%)	5618 (100%)	768 (100%)
Interval between IOP and online test			
<6 months	138 (19%)	129 (19%)	9 (15%)
6 months – 1 year	74 (10%)	69 (10%)	5 (8.2%)
1–3 years	356 (49%)	325 (48%)	31 (51%)
>3 years	164 (22%)	148 (22%)	16 (26%)

Characteristics of the validation population with a refractive error between −3.50D and +2.00D sphere and ≤2.00D cylinder determined by the regulated measurement capability of the device. Ametropia (myopia/hyperopia) was determined by the conventional in-office prescription (IOP).

*D* Diopters, *OD* oculus dexter (right eye), *PDVA* presenting distance visual acuity, *UDVA* uncorrected distance visual acuity.

^a^*n* (%); Mean (SD).

^b^All numbers pertain to seperate monocular measurements, with the exception of sex, age, interval between both refractions, and satistfaction rate.

^c^As measured by the web-based test.

### Refractive error assessment

**Fig. 2:**
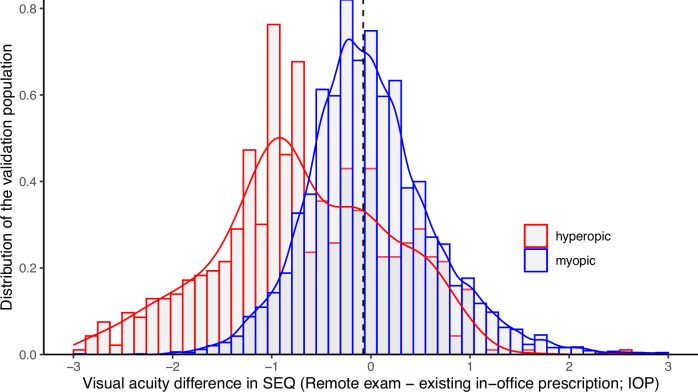
Distribution of refractive errors in the validation population (PDVA ≥ 1.25 decimal Snellen, satisfaction ≥4/5). The dotted line represents the mean difference of the myopics and hyperopics together (mean −0.08D (95% CI −0.10; −0.06)). Y- and x-axis are based on the measurement function of the online test (−3.50 to +2.00D). Hyperopic/myopic determined by the conventional in-office prescription (IOP). SEQ spherical equivalent, PCDVA corrected presenting distance visual acuity, D Diopters.

**Fig. 3:**
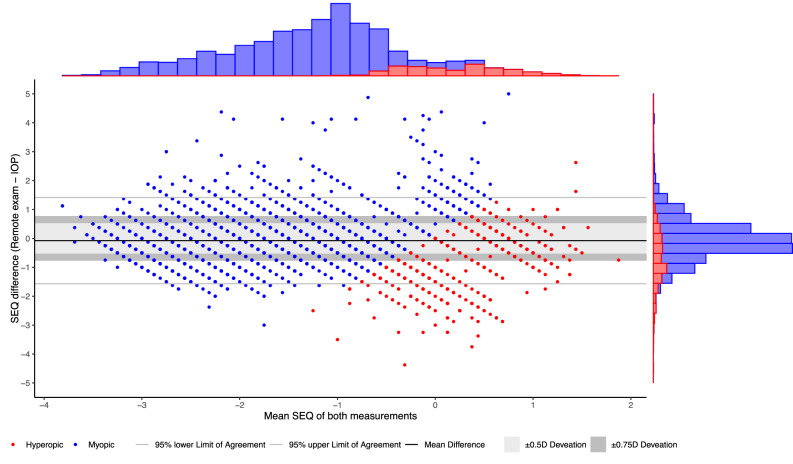
Bland–Altman plot of the SEQ for the validation population (VP; PDVA ≥ 1.25 decimal Snellen, satisfaction ≥4/5). The difference between both tests on the y-axis (remote test – conventional in-office prescription; IOP) is compared to the mean of these measurements. 95% Limits of Agreement (LoA) in D for myopics and hyperopics separate are −1.34;1.37 and −2.53;1.08 lower- and upper limit respectively. Y- and x-axis are based on the measurement function of the online test (−3.50 to +2.00D). Hyperopic/myopic is determined by IOP. SEQ spherical equivalent.

**Fig. 4:**
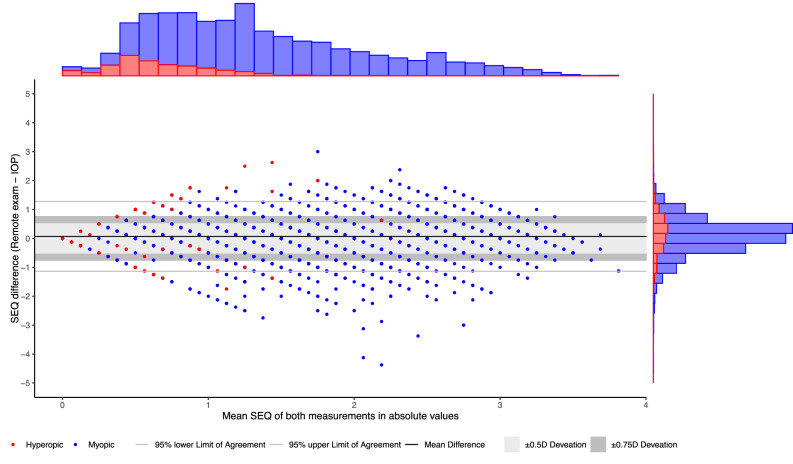
Bland–Altman plot of the SEQ in absolute values of the validation population (VP-Abs; PDVA ≥ 1.25 decimal Snellen, satisfaction ≥4/5). The difference between both tests on the y-axis (remote test – conventional in-office prescription; IOP) is compared to the mean of these measurements. The light gray rectangle represents a deviation of 0.5D, the dark gray rectangle of 0.75D. 95% Limits of Agreement (LoA) for myopics and hyperopics separate are −1.14;1.25 and −1.07;1.43 lower- and upper limit respectively. Y- and x-axis are based on the measurement function of the online test (−3.50 to +2.00D). Hyperopic/myopic is determined by IOP. SEQ spherical equivalent.

The distribution of refractive errors in terms of SEQ is visually reported in Fig. 2; a general underestimation of hyperopia can be appreciated. The refractive mean difference of the VP in SEQ is 0.01D (SD 0.69; 95%LoA −1.34;1.37) and −0.73D (SD 0.92; 95%LoA −2.53;1.08) for myopics and hyperopics respectively. For myopes, 67% of participants are within ±0.5D, and 82% within ±0.75D. For hyperopes, 34% of participants are within ±0.5D, and 50% within ±0.75D. Signation is correctly reported in 96% and 44% of cases in myopes and hyperopes respectively. A post-hoc analysis nullifying the impact of signation errors was based on absolute values (i.e. the VP-Abs group). Then, the mean differences were 0.05D (SD 0.61; 95%LoA −1.14;1.25) and 0.18D (SD 0.64; 95%LoA −1.07;1.43) for myopes and hyperopes respectively, with 70% of myopes were within ±0.5D, and 85% within ±0.75D. For hyperopes, 57% of participants were within ±0.5D, and 80% within ±0.75D. Additional determinants of refractive outcomes are reported in Table 3, power vectors are reported in Supplementary Table 2. These differences are depicted in a Bland–Altman plot for the VP (Fig. 3) and VP-Abs (Fig. 4) respectively.

**Table 3:** Refractive outcomes of the VP-Abs and validation population (VP)

		Refractive components^a^		Mean difference (95% CI)
	Population	IOP	Remote	
Overall (*n* = 6386 eyes)				
SEQ	VP	−1.16 (1.01)	−1.24 (0.98)	−0.08 (−0.10; −0.06)
Sphere		−0.91 (1.01)	−1.07 (1.00)	−0.15 (−0.17; −0.13)
Cylinder	−0.50 (0.54)	−0.49 (0.56)	−0.34 (0.15; 0.13)	−0.34 (0.15;0.13)
Axis		76.87 (65.22)	54.76 (60.39)	−22.11 (−24.05; −20.17)
SEQ	VP-Abs	1.30 (0.82)	1.37 (0.78)	0.07(0.05; 0.08)
Sphere		1.12 (0.78)	1.23 (0.79)	0.11 (0.09; 0.12)
Cylinder		0.57 (0.48)	0.34 (0.26)	−0.23 (−0.25; −0.22)
Axis		76.87 (65.22)	54.76 (60.39)	−22.11 (−24.05; −20.17)
Myopia (*n* = 5618 eyes)				
SEQ	VP	−1.40 (0.80)	−1.38 (0.90)	0.01 (−0.01; 0.03)
Sphere		−1.14 (0.83)	−1.21 (0.93)	−0.07 (−0.09; −0.05)
Cylinder	−0.51 (0.53)	−0.51 (0.54)	−0.34 (0.17; 0.15)	−0.34 (0.17;0.15)
Axis		76.34 (64.92)	54.34 (60.31)	−22.00 (−24.05; −19.94)
SEQ	VP-Abs	1.40 (0.80)	1.45 (0.79)	0.05 (0.04; 0.07)
Sphere		1.17 (0.80)	1.29 (0.81)	0.13 (0.11; 0.15)
Cylinder	−0.32 (0.63)	0.57 (0.47)	0.34 (−0.23; −0.24)	0.34 (−0.23;−0.24)
Axis		76.34 (64.92)	54.34 (60.31)	−22.00 (−24.05; −19.94)
Hyperopia (*n* = 768 eyes)				
SEQ	VP	0.58 (0.53)	−0.15 (0.84)	−0.73 (−0.79; −0.66)
Sphere		0.75 (0.56)	0.02 (0.83)	−0.73 (−0.80; −0.66)
Cylinder		−0.32 (0.63)	−0.35 (0.69)	−0.34 (0.01; −0.04)
Axis		80.76 (67.25)	57.80 (60.89)	−22.95 (−28.79; −17.12)
SEQ	VP-Abs	0.58 (0.53)	0.76 (0.39)	0.18 (0.13; 0.22)
Sphere		0.78 (0.53)	0.73 (0.41)	−0.05 (−0.1; −0.01)
Cylinder		0.59 (0.49)	0.34 (0.24)	−0.25 (−0.29; −0.22)
Axis		80.76 (67.25)	57.80 (60.89)	−22.95 (−28.79; −17.12)

Refractive outcomes of the validation population (VP), and the outcomes reported in absolute values (VP-Abs). Hyperopic/myopic determined by the conventional in-office prescription (IOP).

*SEQ* spherical equivalent, *CI* confidence interval, *Δ* delta, *LL* lower limit, *UL* upper limit, *Remote* online remote test.

^a^Mean (Standard Deviation). VP-Abs = third generation, which is the validation population in absolute values.

### Bias and subgroup analysis

The Bland–Altman plots for all subgroups, not included in the VP, (i.e. either low PDVA or low satisfaction, or both), are reported in Supplementary Figs. 1–3. Mean differences deviate more compared to the VP, which are reported with all other refractive outcomes in Supplementary Table 3. This bias assessment reveals no apparent factors that suggest sampling or reporting bias, justifying case selection for the VP. It must be noted that a suboptimal existing prescription leads to a low PDVA, and that a correct test should therefore be different.

Supplementary Fig. 4 reports the effect of test duration on the outcomes. An excessive long duration was determined as being +2 SD in length, i.e. 22 min or longer. No clinical differences were observed for these VP cases compared to cases within 2 SD. Considering the interval between the online test and the in-office prescription: Supplementary Figs. 5 and 6 report the Bland–Altman plot of participants who performed the test within 1 year after receiving their refractive prescription compared to >3 years, which report no differences. Mean difference for the <1 year subgroup is −0.11D (SD 0.69) compared to −0.12D (SD 0.67) for the >3 year subgroup in SEQ.

## Discussion

This paper reports on the real-world performance and accuracy of a remote web-based eye test in an unsupervised home setting, compared to existing in-office prescriptions (IOP) in the validation population (VP, i.e. high VA and high satisfaction), mirroring previously reported clinical performance.The overall mean difference in SEQ is close to zero (−0.08D, 95% CI −0.10 to −0.06) and shows variation, nonetheless still 67% of myopes were within ±0.5D, and 82% within ±0.75D. For hyperopes, 34% of participants are within ±0.5D, and 50% within ±0.75D. Signation is correctly reported in 96% and 44% of cases in myopes and hyperopes respectively. The online test underestimates hyperopia (mean difference −0.73D, SD 0.92), which can be attributed by the involuntary accommodation reflex of the hyperopic eye and inconsistencies in the signation test^17^. When assessing the refractive results in absolute values (VP-Abs), as a means to nullify the effect of inconsistent signation, in particular hyperopes are assessed with higher accuracy; mean difference of 0.05D (SD 0.61) and 0.18D (SD 0.64) for myopes and hyperopes, respectively. Satisfaction rate of the current refraction, the time to perform the test, or the years between both measurements did not influence outcomes. Extensive bias assessments revealed that case selection (high PDVA, high satisfaction) did not negatively influence our results and was justified, and all subgroups were reported for transparency in the Supplementary Information.

An important remark needs to be made on the natural variation in visual acuity and refractive assessments. When visual function is assessed repeatedly, a noticeable and clinically relevant difference suggested by literature is ±0.15 LogMAR for VA and ±0.5D for refractive errors^21,22^. Though we underline the relevance of these cut-offs on the individual level, literature suggests that the variability (as expressed in 95% limits of agreement) of both are considerably larger. For visual acuity, this is dependent on charts and optotypes used and ranges ±0.14 to ±0.18 in ETDRS, ±0.18 to ±0.24 in Snellen single letter testing, and ±0.33LogMAR using the Snellen line assignment method^23–26^. Alike, conventional manifest refractive assessments are notoriously variable. A considerable body of evidence reveals 95%LoA’s with reported variances up to ±1.0D, well above the commonly accepted ±0.5D. Critical appraisal revealed high risks of bias in the clinical research papers and over 10% of poor outcomes in real world optician sampling^27–34^. Therefore, the online refractive outcomes should be interpreted in the light of aforementioned variance and risk of outliers. In that perspective, the heirin reported variation of ±1.36D (VP) and ±1.20D (VP-abs) are achieved without human overread and included inevitable outliers due to uncontrolled user errors. This does not have to be problematic. For example, in screening, we argue that this arbitrary margin could be treated more liberally considering a world with limited access to care due to long waiting times, increased demand for health care, and alarming rising myopia prevalence, with many of people without access to eye care at all^35^. For example, Gibson reports up to 24% of United States counties lack proper access to optometrists or ophthalmologist services^36^. These individuals might be better off with affordable and scalable eye care. We argue that a ± 0.75D limit of agreements might be a more reasonable and workable deviation limit for ophthalmic refractive measurement methods in general, supported by literature on the precision of objective and subjective refractions^37^.

The results also indicate that myopics perform better compared to hyperopics. When assessing the remote signation test (to determine + or –), we observe the online eye test has difficulties distinguishing hyperopia (44% correct hyperopia, 96% correct myopia). The sign is measured by a combination of the duochrome test and a dedicated near/far questionnaire (Supplementary Table 1)^38^. We hypothesize that the questions asked are not sensitive enough for hyperopics and can be compounded by coexisting astigmatism. Being able to see things closely (myopia) is more easily detected by the test. In contrast, the typical young-adult hyperopic participant involuntarily accommodates, after which they can see both near and far. Normally, should hyperopia be of a large enough magnitude (e.g. >2D) to result in complaints such as dizziness, headaches, it is caught by the questionnaire. Yet, some individuals do not experience these complaints and are misclassified. Increasing the sign-performance of the algorithm for hyperopics is important, underlined by the VP-Abs analysis. This is currently warranted by human graders (trained optometrists) in the commercially available version of the online test. These graders manually review ‘red flags’ and compare them with existing prescriptions; hence signation faults are spotted and corrected, or rejected in case of doubt.

Taking all in consideration, the role of the web-based test is to serve as supplemental to current methods, not intended to replace a comprehensive eye examination, aimed at reducing VI due to URE and providing prescription renewals. A major benefit of a digital solution is that this test is 24/7 accessible, ultimately scalable, and convenient – one only needs a smartphone and a computer. Current methods require trained staff, specialized equipement, mydriatics, or all of the above. Yet, the most striking drawback of the online test is that it is less precise compared to current refractive measures. This may lead to more incorrect prescriptions particularly for those unfamiliar with vision testing or those with more complex refractive errors. In addition user errors are difficult to control, in contrast to an in-office setting where an operater identifies signs of poor cooperation or motivation. Therefore, the online test would benefit from reporting information on performance adequacy, aiding in the interpretation of test scores. Hereby, judicious re-testing of URE will leads to a higher test validity and partly solves the accuracy and outlier problem. Re-testing is very accessible for this online test. From a care demand perspective, individuals who enter the test while they exhibit eye symptoms that necessitate the consultation of an eye care professionals are not identified as an additional burden to care. The incremental value of the online test here is limited, though delay in care is marginal and they should visit an eye care professional in their patient journey at some point.

We previously showed that this online eye test produces comparable outcomes in healthy participants, particularly for myopics, as compared to manifest refractions in clinical conditions^17^. We also demonstrated the supervised use of this test amongst post-cataract patients and in uncontrolled conditions at home in eye patients during the COVID pandemic^20,39^. This paper adds the heterogeneous test performance of the real world, outside of any clinical study. Digital tools lend themselves uniquely well to collect heterogeneous data, better known as Real-world Data (RWD)^40,41^. Analyzing RWD is vital for safe, effective and evidence-based deployment, with acceptance for digital health applications globally^42–44^. This paper supports to the increased societal need for more RWD, to generate innovative treatment approaches in clinicians, policy makers, and health-economists^41,45,46^. Moreover, health care is on the doorstep of a paradigm shift to a more digitized environment^47^. We show that unsupervised eye examinations have the potential to contribute to solutions for burdens we face in the coming decades, such as the increased demands for eyecare and the significant increased prevalence of eye diseases^48^. With the striking increase of myopia as an example with an increased later-in-life likelihood for severe sight-threatening complications by 10–40 times^49,50^. Remote eye care in general could be a valid replacement for in-office assessments for specific care pathways (such as postoperative cataract surgery), a valuable addition when health care is limited (e.g. in pandemics), or applicable for individuals in need of glasses^17,51,52^.

This paper has several strengths. First, RWD is a vital addition to existing controlled clinical data as the studied population is more heterogeneous and a realistic representation of the potential users interested in (web-based) eye testing. Moreover, there is no recruitment nor attrition bias, although the precise motivation of participation remains unknown (e.g. curiosity? vision problems? interested in new glasses? invited by retailers via CRM campaigning?). In addition, the nature of our obligatory data completion is that there are no missing records and an high volume of measurements could be assessed, beyond the feasibility of many clinical studies. Since the web-based test is integrated in e-commerce customer journeys, existing prescriptions were automatically entered in the dataset. Only in case of doubt was the existing prescription manually added by the user.

There are several limitations to consider. Approximately half of the data is included for validation (Fig. 1), which might give the impression of limiting the generalizability of the outcomes. A careful a-priori debate with methodologists (I.S.) and statisticians (L.F. & G.V.) led to reporting the excluded data as well to prevent selection and reporting bias. Our bias analysis underlines that the case selection had a negligible effect on the representativity of the selected population. As mentioned before, the intention for participating in web-based testing varies and was uncontrolled, but we assessed that half of the population had a high presenting VA and a high satisfaction with their existing glasses. Hence, the primary intention is not necessarily a deteriorated VA or the development of an eye condition. In addition, we observe no material distinctions between the validation and the overall population at baseline groups (Tables 1 and 2). Though the study design does not lend itself to draw conclusions regarding the non-VP group, there is no reason identified in the non-VP data for a poorer algorithm-performance compared to the validation subgroup. Yet, absence of evidence is not evidence of absence^53^.

Another limitation is that remote eye measurements are best validated head to head with clinical golden standards; a subjective manifest refraction, or even objective refraction, as was done in the underlying clinical trial^23,34,54^. We acknowledge that the existing prescription from the users own optician, optometrist, or ophthalmologist is a proxy for the above mentioned head to head comparison. Notwithstanding, we tested the accuracy and validity of the existing prescriptions by taking corrected distance visual acuity, and only included prescriptions for validation that yielded VA’s of >1.25 decimal Snellen (20/16). Still, this design causes the limitation where the conditions of the online measurement cannot be ascertained (e.g. test set-up, lighting or distance) and the index and reference measurements are taken at different points in time, theoretically introducing possible chronological bias, albeit that the bias analysis did not reveal any impact from this time-effect. Last, in an ideal study, data on both VA, ophthalmic refraction, subjective experience, and quality of life is collected. Yet due to our real-world study design we are worked with the data available to us.

This paper can be prone to post hoc biases such as spin- and reporting bias, as it is commissioned by the company that hosts and commercializes the web-based test. Therefore, the team invested considerable time and means to minimize this source of post hoc bias. Most importantly, we used the same cut-off points and data-analysis plan in this paper as in the 2019 clinical validation study^17^, a controlled trial using the same web-based eye test, published before this dataset was available. We minimized bias by working with an independent epidemiologist (I.S.), ophthalmologist (S.I.) and two statisticians (L.F. and G.V.) who verified the methods, results, conclusions, and the statistical analysis. Lastly, all analyses on the non-VP sample are redone and reported in the supplementary information, for readers to self-investigate.

Implementing and upscaling web-based vision testing poses challenges. All participants were by default in possession of a smartphone, a screen, and digital skills to perform the test. Studies report on the uptake of the researched eye test in patients with less digital skills, e.g. elderly, where two important conclusions can be drawn. On a positive note, even among elderly, the digital test by itself is feasible to perform, with positive reports by users and home-care nurses^55,56^. However, reaching a digital environment safely is a barrier for many patients. Important factors to consider are that digital proficiency varies considerably from country to country. Seamless integration (*single sign on & API integration*) and a flawless user experience severely reduces drop-off when considering a health care triaging proposition including this web-based eye test^52^.

In conclusion, this study was performed to assess the real-world performance and accuracy of an unsupervised remote web-based eye test in an uncontrolled home-environment of this validated population. This real-world data shows variation, yet still 67% and 82% of measurements were within ±0.5 and ±0.75D. This shows the promise of this technology to increase accessibility to eye care. These analyses also learned that the test underestimates hyperopia due to involuntary accommodation and inconsistencies in the signation test. Bias analyses underline that our case selection for validation purposes was justified. These outcomes are achieved without any interpretation of a human grader, contrary to the commercialized service, where prescriptions are only issued after review by a trained and experienced optometrist.

Future developments should address accuracy and sign determination since this will prove valuable in identifying faulty measurements and reducing overall test variation. This proof-of-concept has the potency of delivering a valuable alternative for uncomplicated in-office refractive assessments. Notwithstanding, possession of a phone and screen, digital skills, and trust in the technology are essential for successful adoption of this remote technology.

## Methods

### Study design and recruitment

Data was collected by Easee B.V., Amsterdam, the Netherlands, the developer of this online eye test. In this study, only the algorithm was researched, not the commercially available platform including human graders (optometrists). All consecutive refractive tests performed between January 1^st^ 2021 and December 31^st^ 2022 were included in this study. All participants were between the ages of 18 and 45 years. Only healthy participants with no self-reported ocular conditions or medical history were eligible to conduct the refraction test. The health questionnaire specifically asked about diabetes, glaucoma, strabismus, previous eye surgery, cataract, eye infections, corneal ulcers, contact lenses, pregnancy, breastfeeding, and individuals with any other eye complaints such as double vision, flashes, floaters, red eye, pain, or irritation. Participants whose refractive error was worse than −3.50 Diopters Spherical (S), +2.00 D or −2.00 Diopters Cylinder (C) were not eligible, as these values constitute the measurement function of the medical device. Effectively, the boundaries regarding both age and refractive error were determined by the regulatory limits of the web-based test.

For comparison and validation purposes, the last measured in-office prescription of participants was used. This prescription was considered a proxy to the current refractive error for people entering the remote eye test. People without an existing prescription, e.g. first time users, were excluded from the dataset. The remote eye test algorithm did not consider the existing prescription in its calculations, however in the commercialized service a human grader manually reviews user performance and a prescription when available. Please note that in this study we reported on algorithm performance only, not on the complete service including manual review by an eye care professional. To prevent post hoc bias, methodological cut-off points for this analysis were duplicated from the 2019 clinical validation study, the MORE trial^17^.

### Web-based refractive eye test

The remote eye test is web-based and is identical to the second-generation algorithm described previously^17,19^. Easee B.V. employs an ISO 13485 Quality Measurement System and the ‘*Easee online eye test’* is classified as *Conformité Européenne* (CE) class 2A medical device according to the Medical Device Regulation 2017/745. Users needed a mobile phone, a second screen (computer or tablet) and three meters (3 m) of distance from the second screen. The phone was paired and functions as a remote control to the screen. Both devices guided the participant through the test with audio and visual instructions. After setup, the computer screen displayed a sequence of optotypes which the participant aimed to identify on their phone. VA was measured both corrected and uncorrected. Participants were explicitly instructed when to wear their refractive correction and which eye to cover. An essential assumption is that any visual acuity below 1.25 decimal Snellen (worse than 20/16 Snellen) was interpreted as resulting from a refractive error. Measuring the appropriate signation (i.e. myopia or hyperopia, − or +) was based on an adapted red/green duochrome test combined with a questionnaire. (see Supplementary Table 1)^38^. Both eyes were tested independently and sequentially, right eye first.

### Statistical analysis

Collected data included age in years, sex, eyewear type, duration of the remote test (in minutes), bilaterality (inclusion of both eyes), satisfaction rate of the current refraction rated from 1 to 5, last previous prescription in spherical and cylindrical power, cylindrical axis, uncorrected and presenting distance visual acuity in logarithm of the minimum angle of resolution (UDVA and PDVA respectively; LogMAR), refractive sign, and the interval between the existing prescription and the online test (in years). The refractive outcomes were converted to power vectors using Fourier analysis^57,58^. A vector is a combined representation of the sphere, cylinder, and axis components. To facilitate straightforward statistical comparison, a residual vector of the difference between the power vectors was calculated.

The primary outcome of performance was defined as the difference in refractive error (sign, spherical and astigmatism power) measured in SEQ between the remote eye test and the participant’s existing prescription. Outcomes were reported as mean differences with 95% confidence intervals, stratified for myopia and hyperopia. Additionally, both methods of refractive assessment were visualized using Bland–Altman plots. The dataset was set up to ensure no missing values could occur. Additional analyses were performed on the length of the eye test (whether the test duration varied more than 2 SD from mean), and whether the interval in years between existing prescription and the online test had effect on the primary outcome. Data was analyzed using R Studio (Comprehensive R Archive Network (CRAN), version 4.3.0).

### Constituting the validation subgroup and bias-analyses

Subgroups were divided by a PDVA of ≥1.25 (*high*; 20/16) or ≤0.8 (*low*; 16/20) decimal Snellen as measured by the online test, in combination with a high (≥4 of 5) and low (≤3) satisfaction rate. We defined PDVA as the visual acuity obtained during the online test, with their current eyeware based on the recorded existing previous prescription. The subgroup of users with a high PDVA and high satisfaction constitute the VP. This was based on the assumption that a young, healthy adult population with no apparent awareness of change in their refractive error should achieve VA ≥ 1.25 decimal Snellen with a valid existing prescription, and that their refraction remained stable over several years^21,54,59^.

The online eye test determined the refraction using three algorithms: the signation assessment (+ or −), the amount of defocus or power error, and the cylinder power and axis. An accurate assessment of all three is needed for an accurate refractive assessment, and all three have their particular impact on measurement variation. For example, a defocus/power error of 0.5D could have been misinterpreted as −0.5D due to an inconsistent signation. Attributing this delta 1D to a defocus/power assessment error would be an overestimation of the variability of this part of the algorithm, since the the signation was off. Therefore, we reported these assessments separately. The signation was analyzed in percentages by comparing the prescription to the online eye test. The defocus/power error was analyzed in a subgroup where the validated population was reported in absolute SEQ values, referred to as ‘VP-Abs’. Vectorized calculations accounted for the impact of astigmatism.

We considered that the measurements for the low PDVA (≤0.8 decimal Snellen, 16/20 Snellen) subgroups had no value as reference measurement in this study design. It is unknown why the presenting VA was sub-par in this group (e.g. faulty conventional prescription, mild amblyopia, lack of compliance). In addition, a considerable amount of time between both measurements (months to years) theoretically introduced chronological bias. In these subgroups with a suboptimal VA, a stable refractive error cannot be assumed since we did not know if, and when, it changed. Nevertheless, the analyses were still supplied for these subgroups as well in the Supplementary Information to prevent reporting bias.

Due to the commercial value of the outcomes of this study to Easee B.V., this paper was potentially prone to spin- and reporting bias. Potential biases were addressed by adhering to a predefined study design commensurate with the 2019 clinical validation MORE trial, and by working with an independent epidemiologist (I.S.), ophthalmologist (S.I.) and two statisticians (L.F. and G.V.) who verified the design, introduction, methods, results, conclusion, dataset, and the statistical analysis.

This study has been performed in accordance with the Declaration of Helskini and EU regulations. Informed consent for data usage was obtained by all participants. Approval by an ethics committee or institutional review board is not applicable since this commercial and retrospective data of Easee B.V. Data is fully anonymized and irreducible.

## Supplementary information

Supplementary Information

## Data Availability

All authors have access to the data in the study and take responsibility for the data integrity and accuracy of the analysis. The data used in this manuscript may be available for research purposes from the corresponding author upon reasonable request.

## Code availability

All authors have access to the code in the study and take responsibility for the integrity and accuracy of the analysis. The code that supports the findings of this study may be available from the corresponding authors upon reasonable request.
