# Supplementary material for: Real world data on digital remote refraction in a healthy population of 14,680 eyes
Source: NPJ Digit Med. 2025 Feb 6;8:89. doi: 10.1038/s41746-025-01453-0 (PMC11802787; doi:10.1038/s41746-025-01453-0)
Supplement: Supplementary file 1 — Supplementary Information [file 41746_2025_1453_MOESM1_ESM.pdf]

Supplementary Information

**Supplementary Table 1. Questions asked during the sign test**
Do you suffer from headaches after doing a lot of reading without glasses? Yes/No
Do you have trouble reading road signs on the highway without your glasses? Yes/No
Do you feel that without glasses you recognize something in the distance later than someone else? Yes/No
Do you suffer from eyestrain after a lot of computer or reading work when you don't wear glasses? Yes/No
Do you feel that letters '*dance*' when reading when you are not wearing glasses? Yes/No
Do you find it difficult to recognize faces in the distance without glasses? Yes/No

Supplementary Table 2. Power vector components of the validation population (VP)

Population	Variables	Power Vector components[1,2]		Mean Difference (95% CI)
		IOP	Remote test	
Overall	Power Vector	1.35 (0.78)	1.34 (0.77)	-0.01 (-0.03;0.00)
	J0	0.05 (0.32)	0.01 (0.17)	
	J45	0.00 (0.17)	0.00 (0.12)	
Myopes	Power Vector	1.46 (0.77)	1.47 (0.78)	0.01 (0.00;0.03)
	J0	0.05 (0.32)	0.01 (0.18)	
	J45	0.00 (0.18)	0.00 (0.12)	
Hyperopes	Power Vector	0.72 (0.49)	0.78 (0.39)	0.06 (0.02;0.10)
	J0	0.06 (0.33)	0.00 (0.17)	
	J45	0.01 (0.19)	0.00 (0.11)	

Power vector outcomes of the validation population of the conventional in-office prescriptions (IOP) and the remote test (PDVA ≥1.25 decimal Snellen, satisfaction ≥4/5). J0 and J45 are the Jackson Cross Cylinder power at 0 and 45 degrees. CI = confidence interval. PDVA = presenting distance visual acuity. Hyperopic/myopic determined by the IOP. [1] mean (SD). [2] Spherical and cylindrical power and axes were translated into vectors using Fourier analysis and the difference is calculated as a power vector of the difference between the power vectors. The difference between power vectors and the vector specific parameters are calculated as a residual vector and is non-linear. [3] mean difference of the refractive components.

Supplementary Table 3. Subgroups with low PDVA and/or low satisfaction

Population		Refractive components[1,2] IOP	Remote	Mean Difference (95% CI)
**Myopic**				
PDVA: high SA: low n=1392	Power Vector	1.31 (0.75)	1.39 (0.76)	0.08 (0.05; 0.11)
	J0	0.05 (0.32)	0.01 (0.19)	
	J45	0.00 (0.17)	0.00 (0.13)	
	SEQ	-1.24 (0.77)	-1.27 (0.92)	-0.03 (-0.07; 0.01)
	sphere	-1.00 (0.79)	-1.09 (0.94)	-0.09 (-0.13; -0.05)
	cylinder	-0.50 (0.55)	-0.36 (0.27)	0.13 (0.10; 0.16)
	axis	76.65 (65.96)	58.45 (61.43)	-18.20 (-22.23; -14.18)
PDVA: low SA: high n=2214	Power Vector	1.49 (0.78)	1.94 (0.84)	0.44 (0.41; 0.47)
	J0	0.08 (0.34)	0.02 (0.20)	
	J45	0.00 (0.19)	0.00 (0.13)	
	SEQ	-1.43 (0.80)	-1.84 (1.00)	-0.41 (-0.45; -0.38)
	Sphere	-1.16 (0.82)	-1.66 (1.03)	-0.50 (-0.53; 0.46)
	Cylinder	-0.53 (0.58)	-0.36 (0.31)	0.17 (0.14; 0.19)
	Axis	76.09 (66.02)	54.72 (60.33)	-21.37 (-24.62; -18.12)
PDVA: low SA: low n=1604	Power Vector	1.34 (0.74)	1.97 (0.85)	0.63 (0.59; 0.66)
	J0	0.07 (0.33)	0.00 (0.20)	
	J45	0.01 (0.18)	0.00 (0.14)	
	SEQ	-1.27 (0.76)	-1.84 (1.07)	-0.57 (-0.62; -0.53)
	Sphere	-1.03 (0.79)	-1.66 (1.09)	-0.62 (-0.67; -0.58)
	Cylinder	-0.48 (0.59)	-0.37 (0.31)	0.10 (0.07; 0.13)
	Axis	71.87 (64.36)	56.88 (59.10)	-14.99 (-18.82; -11.17)
**Hyperopic**				
PDVA: high SA: low n =294	Power Vector	0.65 (0.53)	0.79 (0.50)	0.14 (0.06; 0.21)
	J0	0.05 (0.35)	0.00 (0.17)	
	J45	-0.02 (0.17)	-0.01 (0.12)	
	SEQ	0.52 (0.52)	-0.12 (0.90)	-0.64 (-0.75; -0.53)
	Sphere	0.70 (0.53)	0.05 (0.89)	-0.65 (-0.75; -0.55)
	Cylinder	-0.36 (0.70)	-0.34 (0.24)	0.02 (-0.07; 0.10)
	Axis	79.23 (67.43)	58.45 (60.65)	-20.79 (-30.26; -11.32)
PDVA: low SA: high n=272	Power Vector	0.82 (0.59)	1.11 (0.44)	0.29 (0.21; 0.37)
	J0	0.06 (0.37)	0.00 (0.17)	
	J45	0.00 (0.23)	0.00 (0.12)	
	SEQ	0.67 (0.61)	-0.27 (1.14)	-0.95 (-1.10; -0.80)
	Sphere	0.84 (0.58)	-0.10 (1.13)	-0.95 (-1.09; -0.80)
	Cylinder	-0.34 (0.81)	-0.34 (0.25)	0.00 (-0.10; 0.10)
	Axis	80.38 (66.71)	55.68 (59.84)	-24.7 (-34.16; -15.24)
PDVA: low SA: low	Power Vector	0.74 (0.62)	1.14 (0.51)	0.41 (0.31; 0.50)
	J0	0.01 (0.40)	0.02 (0.18)	

n=258	J45	-0.01 (0.18)	0.00 (0.13)	
	SEQ	0.62 (0.60)	-0.15 (1.22)	-0.78 (-0.92; -0.63)
	Sphere	0.75 (0.57)	0.02 (1.23)	-0.73 (-0.87; -0.58)
	Cylinder	-0.26 (0.84)	-0.36 (0.27)	-0.10 (-0.21; 0.01)
	Axis	67.11 (62.92)	60.08 (62.40)	-7.03 (-16.84; 2.78)

J0 and J45 are the Jackson Cross Cylinder power at 0 and 45 degrees. Δ = delta. SEQ = Spherical Equivalent. IOP = conventional in-office prescription. PDVA = presenting distance visual acuity. SA = satisfaction. Hyperopic/myopic is determined by the IOP. [1] mean (SD). [2] Spherical and cylindrical power and axes were translated into vectors using Fourier analysis and the difference is calculated as a power vector of the difference between the power vectors. The difference between power vectors and the vector specific parameters are calculated as a residual vector and is non-linear.

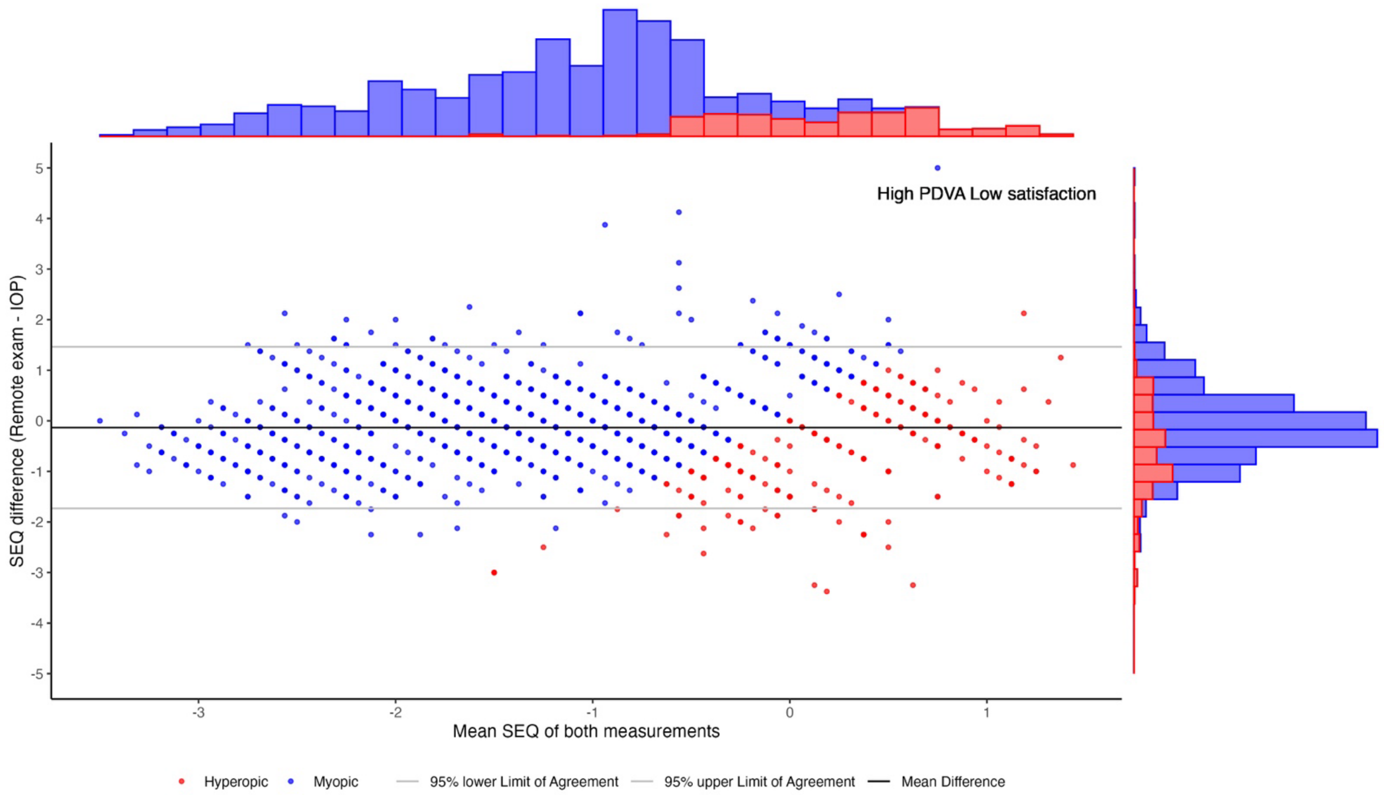

Supplementary Figure 1. Bland-Altman plot of the SEQ for the high PDVA subgroup with low satisfaction (PDVA ≥1.25 decimal Snellen, satisfaction ≤3/5). The difference between both tests on the y-axis (remote test – conventional in-office prescription; IOP) is compared to the mean of these measurements. The black line represents the overall mean difference. The gray lines represent the 95% Limits of Agreement (LoA) of myopes and hyperopes together. LoA's for myopics and hyperopics seperate are -1.46;1.41 and -2.56;1.29D lower- and upper limit respectively. Y- and x-axis are based on the measurement function of the online test (-3.50 to +2.00D). Hyperopic/myopic determined by IOP. SEQ = spherical equivalent. PDVA = presenting distance visual acuity. D = Diopters.

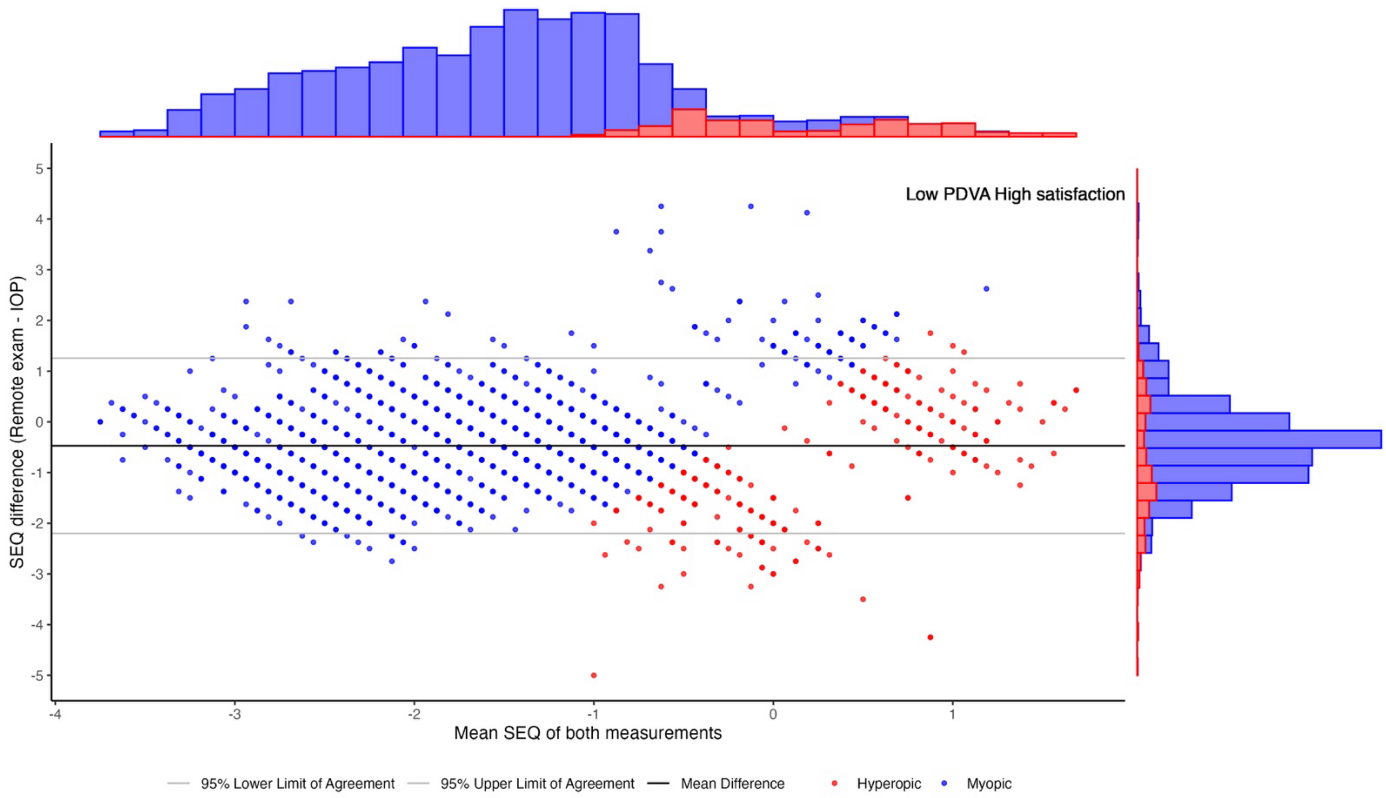

Supplementary Figure 2. Bland-Altman plot of the SEQ for the low PDVA subgroup with high satisfaction (PDVA ≤0.8 decimal Snellen, satisfaction ≥4/5). The difference between both tests on the y-axis (remote test – conventional in-office prescription; IOP) is compared to the mean of these measurements. 95% Limits of agreement (LoA) for myopics and hyperopics seperate are -1.99;1.16 and -3.42;1.52D lower- and upper limit respectively. Y- and x-axis are based on the measurement function of the online test (-3.50 to +2.00D). Hyperopic/myopic determined by IOP. SEQ = spherical equivalent. PDVA = presenting distance visual acuity. D = Diopters.

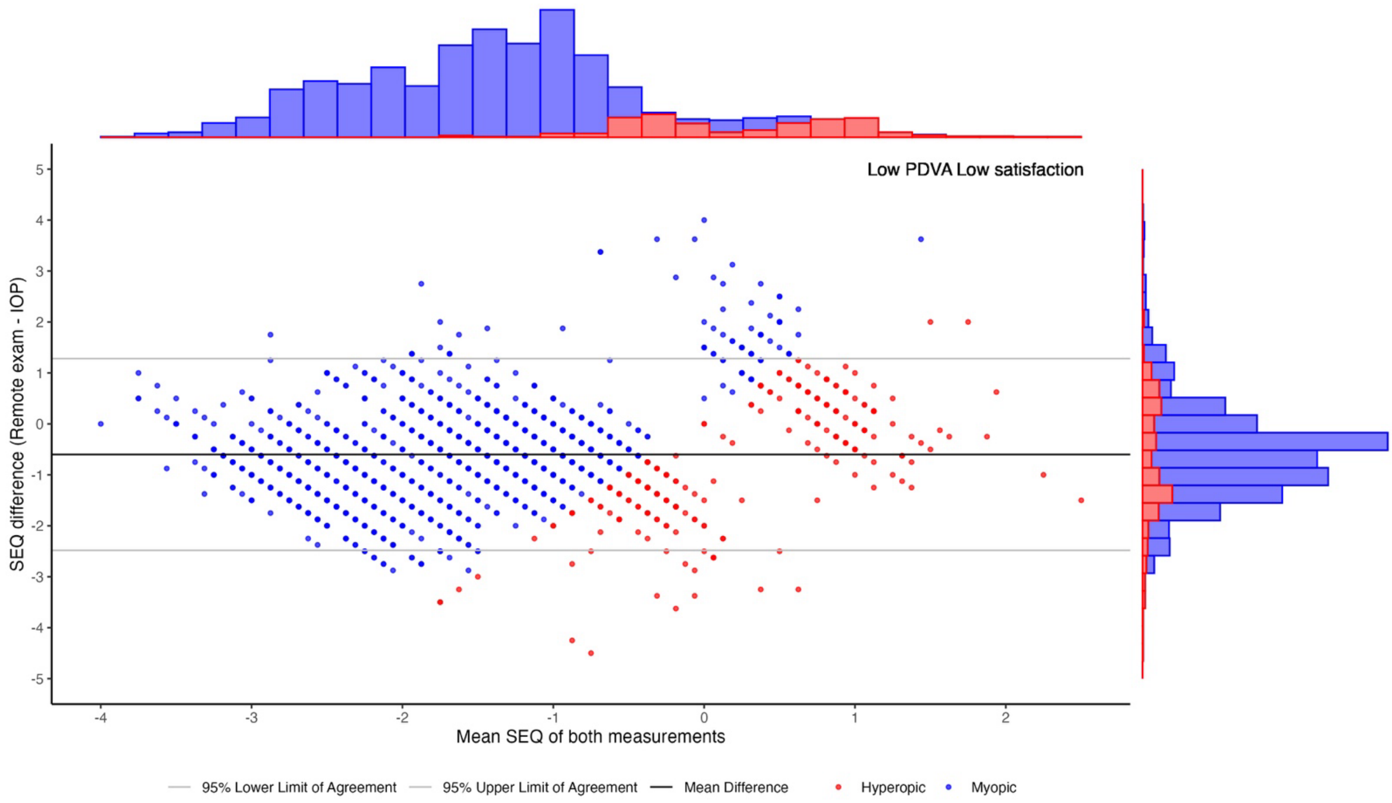

Supplementary Figure 3. Bland-Altman plot of the SEQ for the low PDVA subgroup with low satisfaction (PDVA ≤0.8 decimal Snellen, satisfaction ≤3/5). The difference between both tests on the y-axis (remote test – conventional in-office prescription; IOP) is compared to the mean of these measurements. 95% Limits of Agreement (LoA) for myopics and hyperopics seperate are -2.35;1.21 and -3.15;1.60 lower- and upper limit respectively. Y- and x-axis are based on the measurement function of the online test (-3.50 to +2.00D). Hyperopic/myopic is determined by IOP. SEQ = spherical equivalent. PDVA = presenting distance visual acuity. D = Diopters.

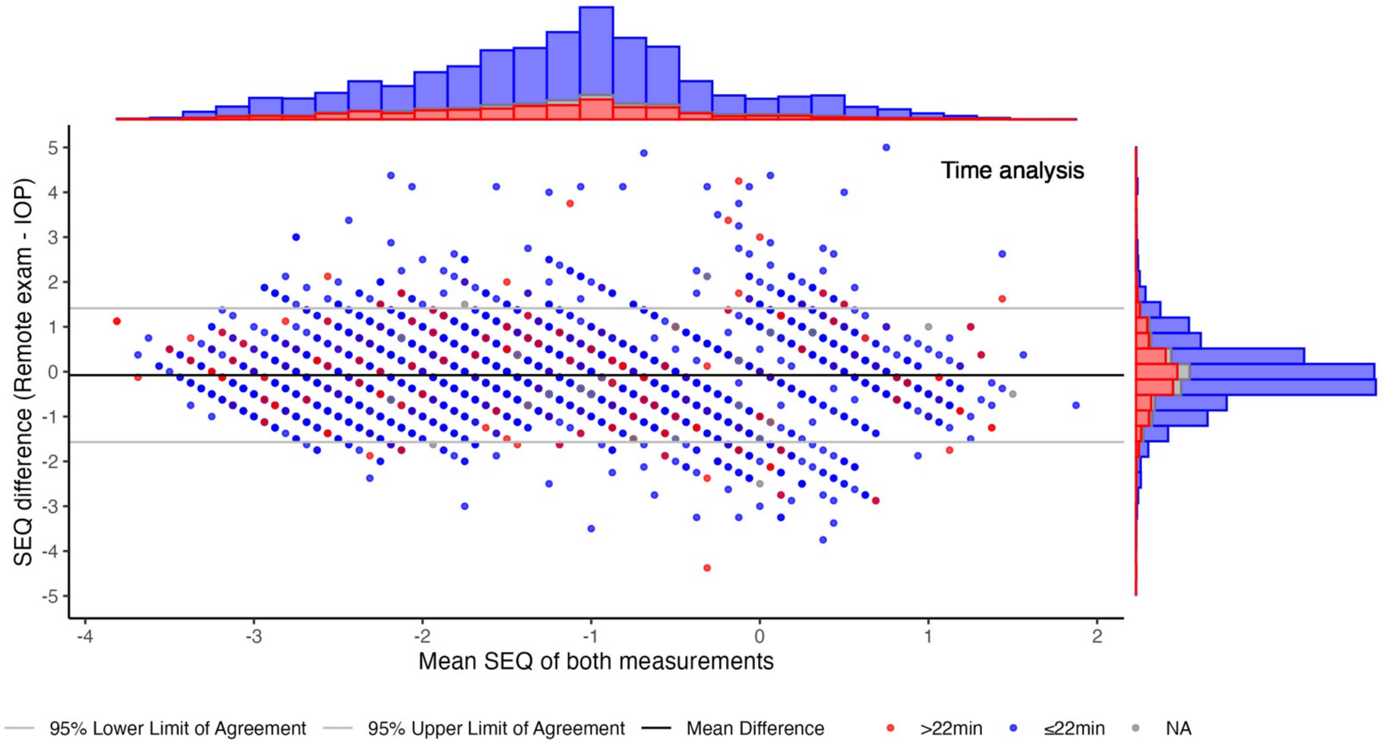

Supplementary Figure 4. Bland-Altman plot of the SEQ for the high PDVA subgroup (PDVA ≥1.25 decimal Snellen, satisfaction ≥4/5) who used over two standard deviations of time to perform the remote test compared to the others compared to the other population (>22 minutes). The difference between both tests on the y-axis (remote test – conventional in-office prescription; IOP) is compared to the mean of these measurements. Y- and x-axis are based on the measurement function of the online test (-3.50 to +2.00D). SEQ = spherical equivalent. PDVA = presenting distance visual acuity. D = Diopters.

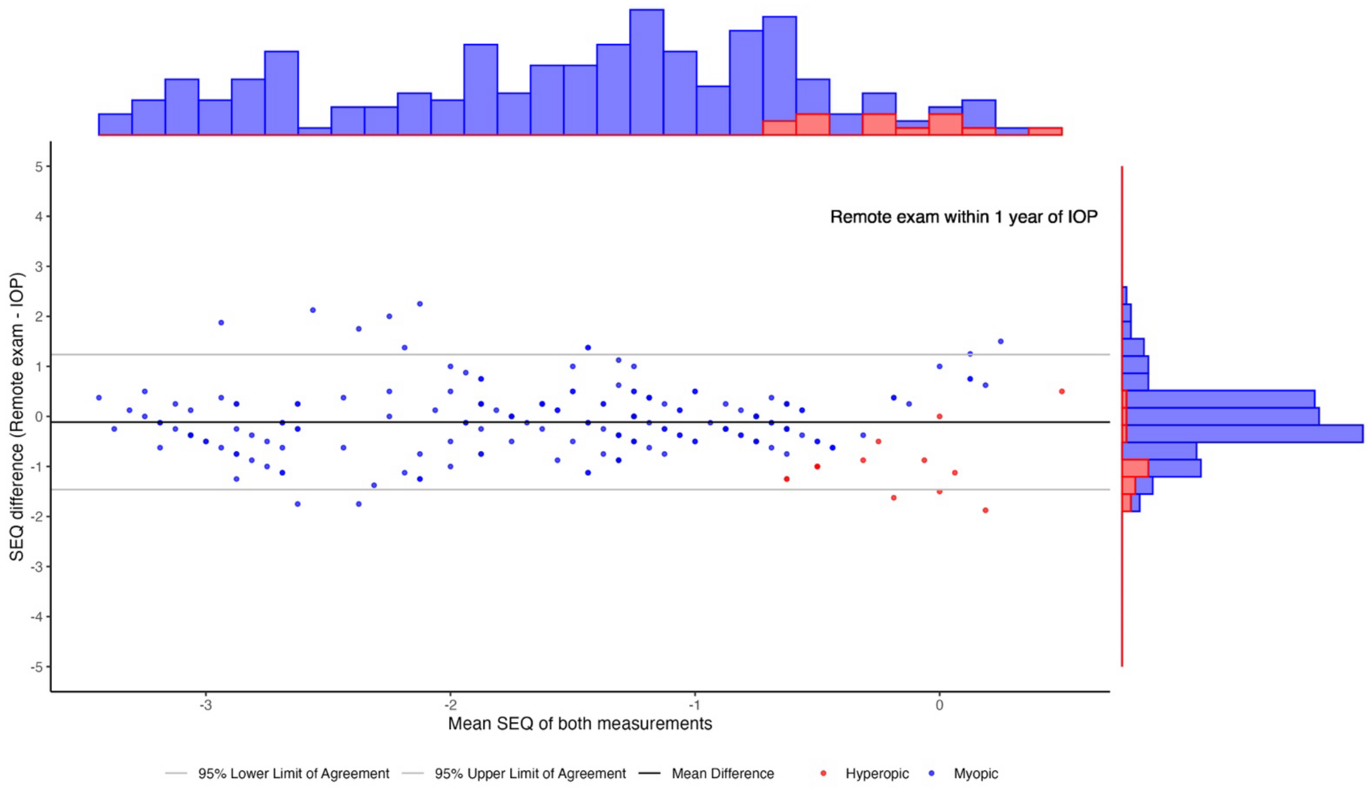

Supplementary Figure 5. Bland-Altman plot of the SEQ for the validation population (PDVA ≥1.25 decimal Snellen, satisfaction ≥4/5) who conducted the test within one year of their last IOP (25% of the participants). The difference between both tests on the y-axis (remote test – conventional in-office prescription; IOP) is compared to the mean of these measurements. Y- and x-axis are based on the measurement function of the online test (-3.50 to +2.00D). Hyperopic/myopic determined by IOP. SEQ = spherical equivalent. PDVA = presenting distance visual acuity. D = Diopters.

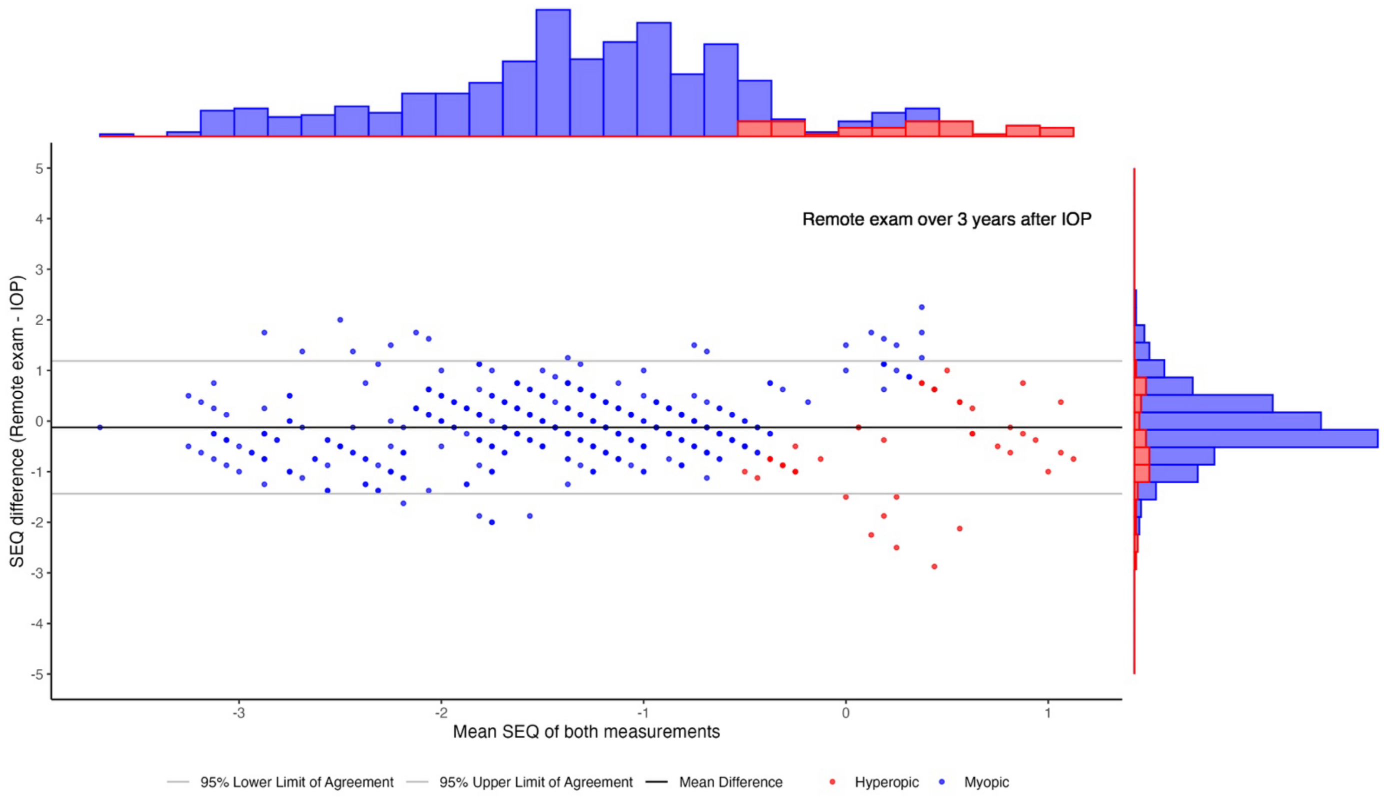

Supplementary Figure 6. Bland-Altman plot of the SEQ for the validation population (P ≥1.25 decimal Snellen, satisfaction ≥4/5) who conducted the test more than three year later than their last IOP (25% of the participants). The difference between both tests on the y-axis (remote test – conventional in-office prescription; IOP) is compared to the mean of these measurements. Y- and x-axis are based on the measurement function of the online test (-3.50 to +2.00D). Hyperopic/myopic determined by IOP. SEQ = spherical equivalent. PDVA = corrected distance visual acuity. D = Diopters.

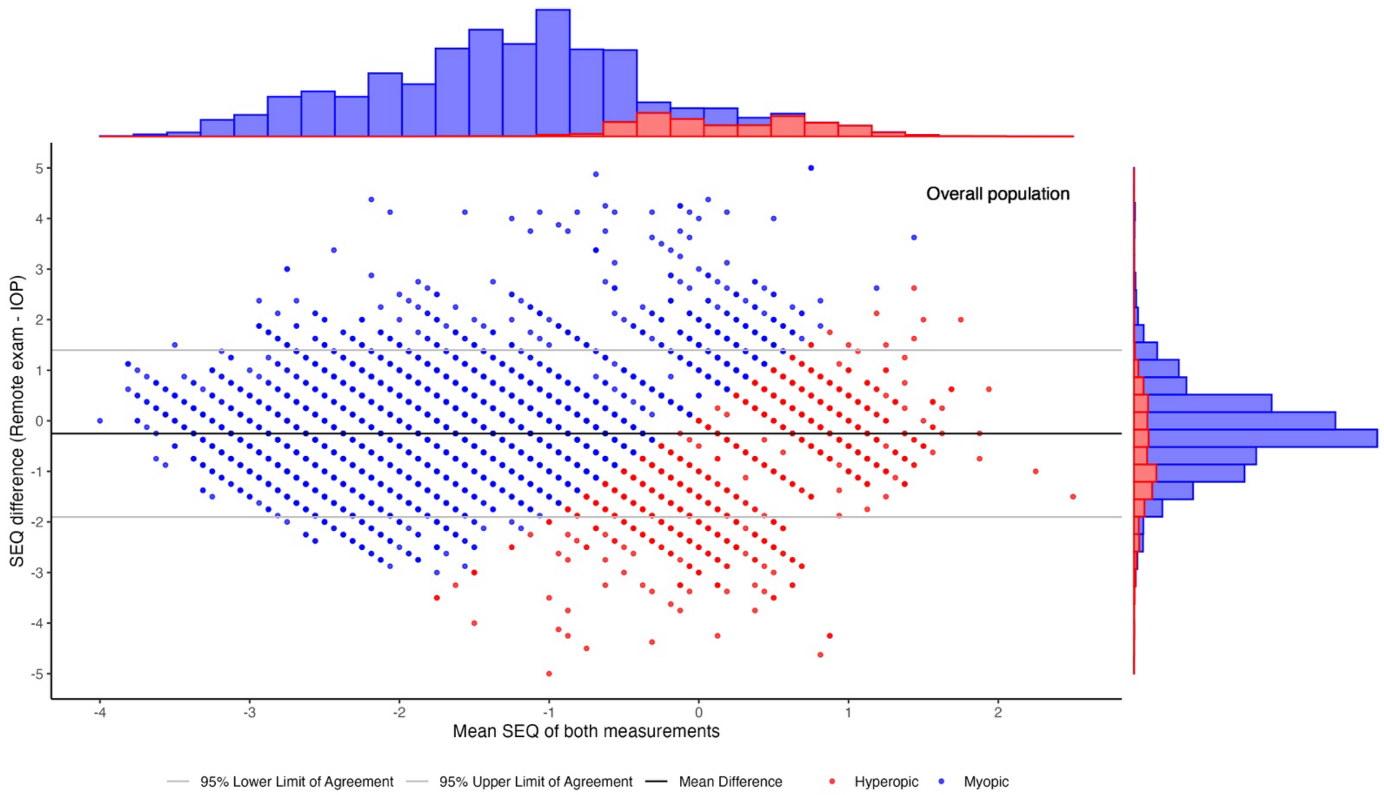

Supplementary Figure 7. Bland-Altman plot of the SEQ for for the overall population (all VA's and satisfaction rates). The difference between both tests on the y-axis (remote test – conventional in-office prescription; IOP) is compared to the mean of these measurements. Y- and x-axis are based on the measurement function of the online test (-3.50 to +2.00D). Hyperopic/myopic is determined by in-office prescriptions. SEQ = spherical equivalent. PDVA = presenting distance visual acuity. D = Diopter.
